# Associations between estimated glucose disposal rate and arterial stiffness and mortality among US adults with non-alcoholic fatty liver disease

**DOI:** 10.3389/fendo.2024.1398265

**Published:** 2024-05-08

**Authors:** Junting Song, Ruicong Ma, Lin Yin

**Affiliations:** ^1^ Department of Neurology, The Second Hospital of Dalian Medical University, Dalian, Liaoning, China; ^2^ Department of Cardiology, The Second Hospital of Dalian Medical University, Dalian, Liaoning, China

**Keywords:** insulin resistance, non-alcoholic fatty liver disease, estimated glucose disposal rate, arterial stiffness, mortality, NHANES

## Abstract

**Background:**

The estimated glucose disposal rate (eGDR), an effective indicator of insulin resistance, has been related to acute coronary syndrome, ischemic stroke and heart failure. This study aims to explore the relationship between eGDR and arterial stiffness, all-cause mortality and cardiovascular mortality in patients with non-alcoholic fatty liver disease (NAFLD).

**Methods:**

Participants with NAFLD were chosen from the National Health and Nutrition Examination Survey (NHANES) between 1999 and 2018. The main outcomes are arterial stiffness (represented by estimated pulse wave velocity, ePWV), all-cause and cardiovascular mortality. Multiple cox regression models, restricted cubic spline, sensitivity analysis and subgroup analysis were carried out to investigate the correlation between the insulin resistance indicators and mortality and arterial stiffness. Furthermore, receiver operating characteristic curves were used to compare the predictive value of the eGDR with the triglyceride-glucose (TyG) index and the homeostasis model assessment of insulin resistance (HOMA-IR) for all-cause and cardiovascular mortality.

**Results:**

In this study, a total of 4,861 participants were included for analysis. After adjusting confounding factors in the multivariate weighted cox regression model, the eGDR was inversely associated with the all-cause mortality (Q4 vs. Q1, HR =0.65 (0.48-0.89, P=0.01) and cardiovascular mortality (Q4 vs. Q1, HR =0.35 (0.19-0.65, P<0.001). Compared with TyG index and HOMA-IR, the eGDR shows excellent predictive value in all-cause mortality (0.588 vs. 0.550 vs. 0.513, P < 0.001) and cardiovascular mortality (0.625 vs. 0.553 vs. 0.537, P < 0.001). In addition, we found a significant negative correlation between eGDR and arterial stiffness (β=-0.13(-0.14–0.11, P< 0.001). However, TyG index and HOMA-IR showed no significant correlation to arterial stiffness.

**Conclusions:**

Low eGDR (an indicator of insulin resistance) levels are related to an increased risk of arterial stiffness and mortality in NAFLD patients in the United States.

## Introduction

Non-alcoholic fatty liver disease (NAFLD) is a common chronic liver disease characterized by abnormal accumulation of fat in the liver. Furthermore, NAFLD does not involve viruses, alcohol, or autoimmune factors. NAFLD (1), accounting for approximately one-third of the population globally, has brought substantial economic and medical burden (2). Moreover, the burden of fatty liver disease is rapidly growing in every region of the world over the past years (3). What is particularly concerning is the rising incidence of NAFLD among younger age groups (4). Approximately 20% of patients with NAFLD will progress to metabolic dysfunction-associated steatohepatitis which can increase the risk of developing liver cirrhosis in the future (5). Additionally, cardiovascular disease (CVD) is the main cause of death in individuals with NAFLD, further highlighting its significant impact on individuals and societies (6). The incidence of cardiovascular adverse events is higher in NAFLD patients, including stroke and myocardial infarction (7, 8). In spite of effective efforts in NAFLD prevention and treatment, managing NAFLD remains challenging. Consequently, evaluating the prognosis of NAFLD patients holds immense importance in the field of public health.

Insulin resistance (IR) is a pathological state in which the body’s sensitivity to insulin decreases (9). IR plays an important role in NAFLD and cardiovascular disease. Some studies have shown that IR promotes the generation of liver fat, which is closely related to the onset and progression of NAFLD (10). In addition, IR is involved in the development of atherosclerosis, hypertension, heart failure and other cardiovascular diseases (11). Although the gold standard for assessing IR is the euglycemic hyperinsulinemic clamp, the clinical utility is limited due to the invasive and costly nature (12). At present, the widespread utilization of the homeostasis model assessment of insulin resistance (HOMA-IR) has been observed. However, it has certain limitations for patients receiving insulin therapy. Therefore, the estimated glucose disposal rate (eGDR) (13)and the triglyceride-glucose (TyG) index (14) have been developed for clinical application.

The eGDR was initially created as a validated measure to assess IR in individuals with type 1 diabetes (T1D) according to hypertension, waist circumference (WC) and glycated hemoglobin A (HbA1c) (15). In comparison to the euglycemic hyperinsulinemic clamp, this technique offers increased accuracy and is suitable for large-scale clinical research (16). Some studies have found that low eGDR is associated with the increased risk of prevalence and poor prognosis in various diseases, such as fatty liver disease, acute coronary syndrome, heart failure and stroke (17–20).

The association between eGDR and NAFLD outcomes is still not well understood, despite its close relationship with many diseases. This study aims to explore the relationship between eGDR and arterial stiffness (represented by estimated pulse wave velocity, ePWV), all-cause mortality and cardiovascular mortality in patients with NAFLD.

## Materials and methods

### Data source and study participates

**Figure 1:**
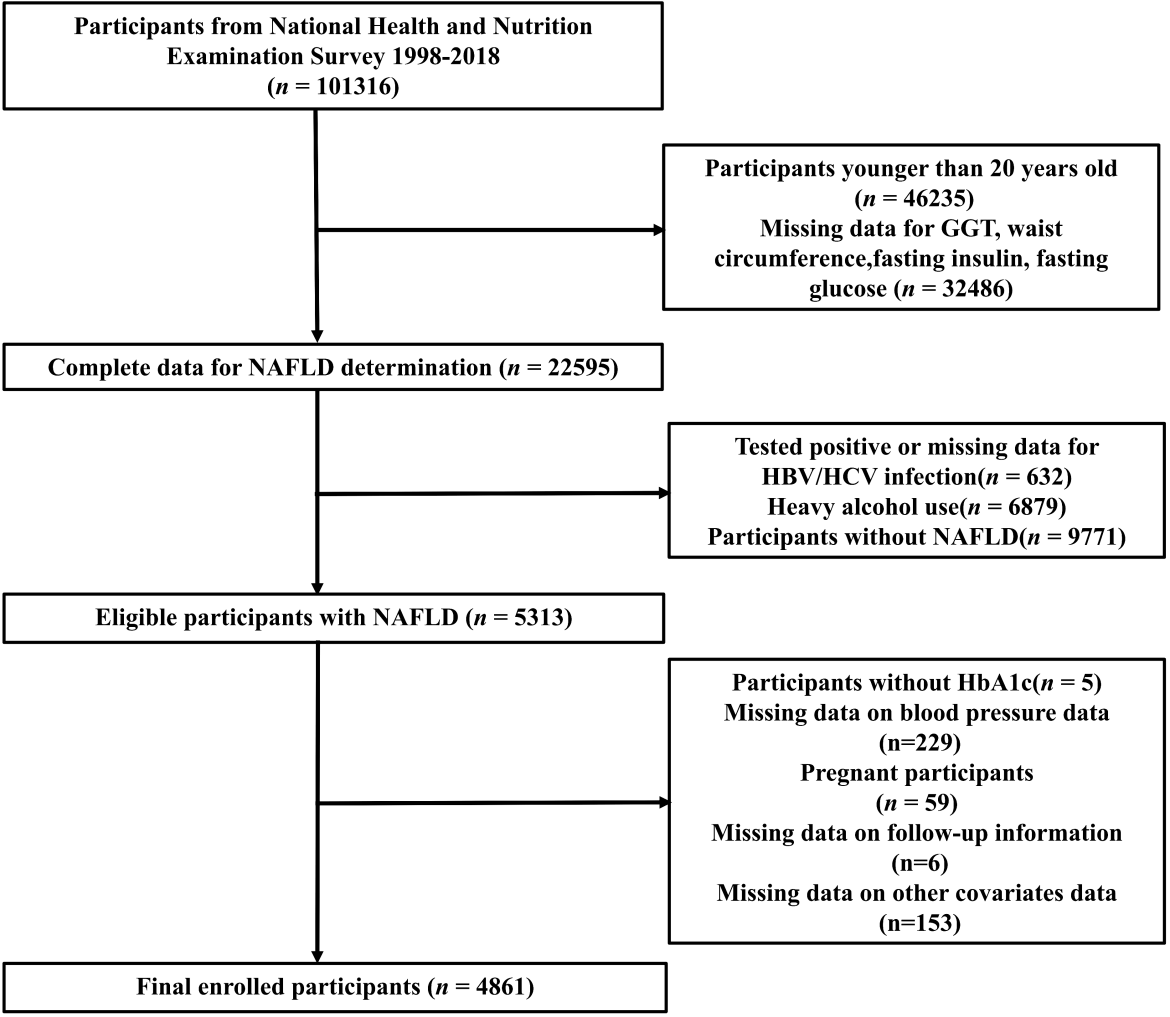
The flow chart of participant selection.

We carried out our study by utilizing data from the National Health and Nutrition Examination Survey (NHANES) database available at www.cdc.gov/nchs/nhanes.com. The purpose was to evaluate the health conditions of individuals aged 20 and older in the United States. The data sets were gathered from various states and counties across the nation. These samples were obtained from all NHANES participants from 1999 to 2018 (n = 101306), we excluded participants whom younger than 20 years (n =46235), those missing data for GGT, waist circumference, fasting insulin or fasting glucose (n = 32486), Participants with tested positive or missing data for HBV/HCV infection(n = 632) and heavy alcohol use(n = 6879), participants without NAFLD (n = 9771), those without HbA1c(n = 5), blood pressure data (n = 229), pregnant participants (n = 59) and participants missing data on follow-up information (n = 6) and other covariates data (n = 153). The analysis sample comprised 4861 participants in total. The screening process details were illustrated in **Figure 1**.

### Diagnosis of NAFLD

The diagnosis of NAFLD usually involves detecting liver fat through imaging examinations such as abdominal ultrasound and magnetic resonance imaging. Additionally, we need to exclude other clear factors of liver injury. If necessary, further liver biopsy is also required. These methods require high operational requirements and are costly. Moreover, steatosis can only be detected when the steatosis rate of liver cells exceeds 20%-30%, it has not been widely applied. Therefore, a score for assessing fatty liver disease in the United States population was developed by CE Ruhl (21). Therefore, we used us-FLI≥30 as a criterion for diagnosing NAFLD.

### Calculation of IR indicators and ePWV

The eGDR (mg/kg/min) was created as a measure of IR and calculated by using the following formula: eGDR = 21.158 − (0.09∗WC) − (3.407∗HT)− (0.551∗HbA1c) [WC = waist circumference (cm), HT = hypertension (yes = 1/no = 0) and HbA1c = HbA1c (%)] (15). In 2008, TyG index was introduced as a reliable and specific predictor of IR. It has been shown to have a good correlation with the hypoglycemic-hyperinsulinemic clamp test and HOMA-IR. The TyG index was calculated as Ln [fasting triglycerides (mg/dL) × Fasting glucose (mg/dL)/2] (22). HOMA-IR is an indicator used to evaluate an individual’s IR level but it is expensive. The HOMA-IR was calculated as fasting insulin (μU/mL) × fasting plasma glucose (mg/dL)/405 (23). We used ePWV to evaluate arterial stiffness. According to the equation, ePWV was calculated from age and mean blood pressure (MBP): 9.587 − 0.402 × age + 4.560 × 10^−3^ × age^2^ − 2.621 × 10^−5^ × age^2^ × MBP + 3.176 × 10^−3^× age × MBP − 1.832 × 10^−2^ × MBP. MBP was calculated as diastolic blood pressure+0.4 × (systolic blood pressure − diastolic blood pressure) (24).

### Covariates

In this study, we selected covariates related to NAFLD based on previous research. Demographic information was obtained from the NHANES database, which contained data on age (in years), sex (categorized as male or female), racial/ethnic background (including white, black, Mexican and others), educational attainment (categorized as less than high school, high school, and post-high school education). This information was obtained from the NHANES demographic questionnaire. Body mass index (BMI) was calculated by dividing weight [kg] by the square of height [m²]. We obtained smoking status (yes/no) from the questionnaire. Coronary heart disease (CHD) and congestive heart failure (CHF) were diagnosed based on medical history. In addition, we collected glycated hemoglobin (HbA1c) (%), total cholesterol (TC) (mmol/L), triglycerides (TG) from laboratory examination data. We calculated the estimated glomerular filtration rate (eGFR) based on the creatinine data of participants provided by NHANES using the Chronic Kidney Disease Epidemiology Collaboration (CKD-EPI) method (25). Hypertension was diagnosed based on guidelines provided by the Joint National Committee on Prevention, Detection, Evaluation, and Treatment of High Blood Pressure. We applied hypertension assessment criteria: SBP ≥ 140 mmHg or DBP ≥ 90 mmHg and the patients using anti-hypertensive medications for the period of being investigated (26). We applied diabetes evaluation criteria: doctor diagnosis as diabetes, HbA1c ≥ 6.5%, fasting glucose ≥ 7.0mmol/L, random blood glucose ≥ 11.1mmol/L, 2h OGTT blood glucose ≥ 11.1mmol/L, or being treated with diabetes drugs and insulin (27).

### Mortality

To assess the mortality, we paired the National Death Index data with the mortality information for the period ending on December 31, 2019 (https://www.cdc.gov/nchs/data-linkage/mortality.htm). Outcomes were be defined as all-cause and cardiovascular mortality. Causes of death were defined according to the codes of ICD-10. Cardiovascular mortality was defined using ICD-10 codes 100-109,111,113,120-151 (28).

### Statistical analysis

Firstly, we divided the data into four groups according to the quartile of eGDR. Continuous variables were presented as means (95% confidence intervals (CI)) and proportions with their respective 95% CI were employed for categorical variables. In order to ascertain variations between the four groups, the variance analysis or Kruskal–Wallis test were conducted for continuous variables, while chi-square tests were utilized for categorical variables. Statistical significance was considered at *P* values < 0.05. We excluded other missing variables after obtaining the main data for the study. Due to the small number of missing variables, we excluded them to ensure the objectivity and accuracy of the results. Finally, the analysis sample comprised 4861 participants in total. When conducting various statistical analyses, we adjusted for demographic variables, hematological indicators and medication information which may have an impact on the prognosis of NAFLD patients (29). Next, we conducted weighted linear regression analyses in order to examine the correlation between eGDR and ePWV. Restricted cubic splines are an important tool in statistics used for smooth fitting and modeling of data, as well as analyzing complex relationships between continuous variables. To examine the correlation between eGDR and ePWV, we employed a restricted cubic spline method. In the multivariate cox regression model, other confounding factors are adjusted so that the real effect can be displayed. Therefore, weighted cox regression analyses were used to investigate the relationship between eGDR and all-cause mortality and cardiovascular mortality. We constructed two models: Model I and Model II. Model I was adjusted for age, sex, race. Model II was adjusted for age, sex, race/ethnicity, education levels, smoking, BMI, TC, TG, eGFR, DM, CHD, CHF, hyperlipidemia, anti-diabetic drugs and anti-hyperlipidemic drugs. Results were presented as hazard ratios (HRs) with 95% CIs. Restricted cubic spline method was used for the correlation between eGDR and mortality. To further ensure the robustness and credibility of the results, sensitivity analysis and subgroup analysis have been adopted in many studies (30). Furthermore, different researchers have utilized different approaches for analysis, such as weighted and unweighted methods. NHANES uses complex sampling techniques to enhance the accuracy and relevance of results. However, discrepancies may arise between weighted and unweighted analyses. Therefore, we conducted a sensitivity analysis using unweighted regression to verify our findings. Receiver operating characteristic (ROC) curve chart is a graph used to evaluate the performance of diagnostic systems and find the optimal threshold. Consequently, we used ROC curves to compare the predictive value of the eGDR with the triglyceride-glucose (TyG) index and the homeostasis model assessment of insulin resistance (HOMA-IR) for all-cause and cardiovascular mortality. Finally, we aim to further clarify the relationship between eGDR and NAFLD in different subgroups.

## Results

### The baseline characteristics of participants

A total of 4.861 participants with NAFLD were involved in the study. As showed in **Table 1**, we divided the data into four groups according to the quartile of eGDR. The baseline characteristics of all participants, including age, sex, race, education levels, smoking, BMI, waist circumference, HbA1c, TG, TC, HOMA_IR, TyG, ePWV, eGFR, DM, CHD, CHF, hyperlipidemia, anti-diabetic drugs, anti-hyperlipidemic drugs, all-cause mortality and cardiovascular mortality are presented in **Table 1**. **Table 1** shows significant differences in clinical characteristics between the four groups. Compared with the lower eGDR group, patients with higher eGDR were younger, higher levels of education, fewer white people. The high eGDR group has fewer smokers, a lower proportion of hyperlipidemia, CHD, CHF and lower use of hypoglycemic and lipid-lowering drugs. Participants with higher eGDR had lower BMI, WC, ePWV, TyG index, HOMA-IR, HbA1c, higher TC and eGFR (P < 0.001). Additionally, both all-cause mortality and cardiovascular mortality significantly decrease as eGDR increases.

**Table 1:** Clinical characteristics of study population grouped by eGDR quartiles.

Variables	Overall	eGDR-Q1	eGDR-Q2	eGDR-Q3	eGDR-Q4	*P* value
Age, %	54.69 (54.10,55.28)	57.17 (56.22,58.12)	59.66 (58.73,60.58)	54.81 (53.60,56.02)	47.50 (46.38,48.63)	<0.001***
Gender, %						0.29
Female	43.01 (39.83,46.20)	42.29 (38.48,46.09)	45.51 (41.77,49.25)	43.98 (39.66,48.30)	40.39 (36.83,43.94)	
Male	56.99 (52.93,61.04)	57.71 (53.91,61.52)	54.49 (50.75,58.23)	56.02 (51.70,60.34)	59.61 (56.06,63.17)	
Race/ethnicity, %						<0.001***
White	72.93 (66.69,79.18)	74.37 (70.99,77.75)	77.16 (74.06,80.25)	73.89 (70.80,76.98)	66.64 (62.87,70.41)	
Black	5.94 (5.20, 6.69)	10.17 (8.10,12.23)	6.30 (4.95, 7.66)	4.95 (3.89, 6.01)	2.57 (1.84, 3.30)	
Mexican	9.93 (8.46,11.39)	5.67 (4.30, 7.04)	6.89 (5.17, 8.60)	9.11 (7.25,10.97)	17.63 (14.81,20.46)	
Others	11.19 (9.83,12.55)	9.79 (7.43,12.15)	9.65 (7.72,11.58)	12.05 (10.19,13.91)	13.16 (10.48,15.84)	
Education levels, %						0.04*
Less than high school	21.63 (19.57,23.69)	20.19 (17.25,23.14)	22.37 (19.29,25.44)	21.01 (18.04,23.98)	22.88 (19.70,26.05)	
High school or equivalent	24.57 (22.01,27.13)	26.32 (22.96,29.68)	27.13 (23.82,30.45)	24.89 (21.41,28.37)	20.16 (17.11,23.22)	
College or above	53.81 (49.87,57.74)	53.49 (49.18,57.79)	50.50 (46.65,54.35)	54.11 (49.96,58.25)	56.96 (53.03,60.89)	
BMI, kg/m2	33.88 (33.58,34.18)	39.76 (39.23,40.30)	33.11 (32.73,33.50)	32.66 (32.20,33.12)	30.22 (29.88,30.55)	<0.001***
waist circumference, cm	113.03 (112.36,113.70)	127.40 (126.35,128.45)	111.91 (111.14,112.69)	110.42 (109.30,111.55)	103.02 (102.27,103.77)	<0.001***
HbA1c, %	6.05 (6.00,6.10)	6.86 (6.73,6.99)	5.99 (5.93,6.05)	5.83 (5.76,5.91)	5.54 (5.50,5.58)	<0.001***
TG, mmol/L	2.03 (1.96,2.10)	2.07 (1.96,2.18)	1.99 (1.89,2.09)	2.01 (1.88,2.15)	2.05 (1.90,2.21)	0.78
TC, mmol/L	5.08 (5.03,5.13)	4.86 (4.78,4.94)	5.04 (4.95,5.12)	5.13 (5.05,5.22)	5.26 (5.15,5.36)	<0.001***
HOMA_IR	7.40 (7.07,7.73)	10.80 (9.88,11.71)	6.86 (6.30, 7.43)	6.63 (6.18, 7.07)	5.43 (5.07, 5.80)	<0.001***
TyG	7.50 (7.47,7.52)	7.68 (7.62,7.74)	7.48 (7.44,7.53)	7.44 (7.39,7.49)	7.38 (7.34,7.43)	<0.001***
ePWV	9.00 (8.92,9.07)	9.35 (9.24,9.47)	9.74 (9.60,9.88)	9.03 (8.87,9.18)	7.93 (7.81,8.05)	<0.001***
eGFR, mL/min/1.73 m^2^	87.32 (86.50,88.15)	84.80 (83.17,86.42)	81.86 (80.41,83.31)	87.99 (86.49,89.50)	94.27 (92.86,95.68)	<0.001***
Smoking, %						0.005**
No	54.35 (50.77,57.94)	52.14 (48.18,56.10)	50.36 (46.61,54.11)	55.56 (51.59,59.52)	59.09 (55.51,62.67)	
Yes	45.65 (41.77,49.52)	47.86 (43.90,51.82)	49.64 (45.89,53.39)	44.44 (40.48,48.41)	40.91 (37.33,44.49)	
DM, %						<0.001***
No	67.43 (62.71,72.15)	40.52 (36.57,44.47)	66.25 (62.97,69.53)	75.02 (71.80,78.24)	86.69 (84.33,89.05)	
Yes	32.57 (30.15,34.99)	59.48 (55.53,63.43)	33.75 (30.47,37.03)	24.98 (21.76,28.20)	13.31 (10.95,15.67)	
CHD, %						<0.001***
No	92.39 (86.76,98.02)	88.56 (85.75,91.38)	91.37 (89.42,93.31)	92.58 (90.52,94.64)	96.79 (95.48,98.11)	
Yes	7.61 (6.42, 8.80)	11.44 (8.62,14.25)	8.63 (6.69,10.58)	7.42 (5.36, 9.48)	3.21 (1.89, 4.52)	
Hyperlipidemia, %						0.10
No	10.95 (9.59,12.32)	10.14 (7.97,12.31)	8.91 (6.72,11.11)	11.87 (9.21,14.53)	12.79 (10.39,15.18)	
Yes	89.05 (83.37,94.72)	89.86 (87.69,92.03)	91.09 (88.89,93.28)	88.13 (85.47,90.79)	87.21 (84.82,89.61)	
CHF, %						<0.001***
No	94.99 (89.17,100.81)	90.62 (88.60,92.64)	94.50 (92.96,96.04)	96.31 (95.02,97.60)	98.32 (97.49,99.14)	
Yes	5.01 (4.19, 5.84)	9.38 (7.36,11.40)	5.50 (3.96, 7.04)	3.69 (2.40, 4.98)	1.68 (0.86, 2.51)	
Anti-diabetic drugs, %						<0.001***
No	80.54 (75.24,85.84)	59.88 (55.93,63.83)	80.75 (78.11,83.39)	87.34 (84.95,89.73)	93.33 (91.42,95.24)	
Yes	19.46 (17.67,21.25)	40.12 (36.17,44.07)	19.25 (16.61,21.89)	12.66 (10.27,15.05)	6.67 (4.76, 8.58)	
Anti-hyperlipidemic drugs, %						<0.001***
No	68.11 (63.62,72.59)	56.97 (53.42,60.52)	59.31 (55.82,62.79)	71.82 (68.08,75.55)	83.45 (80.34,86.56)	
Yes	31.89 (29.22,34.56)	43.03 (39.48,46.58)	40.69 (37.21,44.18)	28.18 (24.45,31.92)	16.55 (13.44,19.66)	
All-cause mortality, %						<0.001***
No	83.15 (77.82,88.48)	79.23 (76.19,82.27)	80.07 (77.31,82.83)	81.68 (79.04,84.32)	91.21 (89.32,93.09)	
Yes	16.85 (15.15,18.55)	20.77 (17.73,23.81)	19.93 (17.17,22.69)	18.32 (15.68,20.96)	8.79 (6.91,10.68)	
Cardiovascular mortality, %						<0.001***
No	95.40 (89.44,101.37)	93.44 (91.63,95.26)	93.95 (92.33,95.58)	95.43 (94.03,96.83)	98.61 (97.91,99.31)	
Yes	4.60 (3.86, 5.33)	6.56 (4.74,8.37)	6.05 (4.42,7.67)	4.57 (3.17,5.97)	1.39 (0.69,2.09)	

Continuous data were presented as the mean and 95% confidence interval, category data were presented as the proportion and 95% confidence interval. eGDR, estimated glucose disposal rate; BMI, body mass index; HbA1c, glycosylated hemoglobin; TC, total cholesterol; TG, triglycerides; HOMA-IR, Homeostatic Model Assessment for Insulin Resistance; TyG, triglyceride and glucose index; ePWV, estimated pulse wave velocity; eGFR, estimated glomerular filtration rate; DM, diabetes mellitus; CHD, coronary heart disease; CHF, congestive heart failure; ***P value<0.001, **P value<0.01, *P value<0.05.

### Relationship between eGDR and arterial stiffness

**Figure 2:**
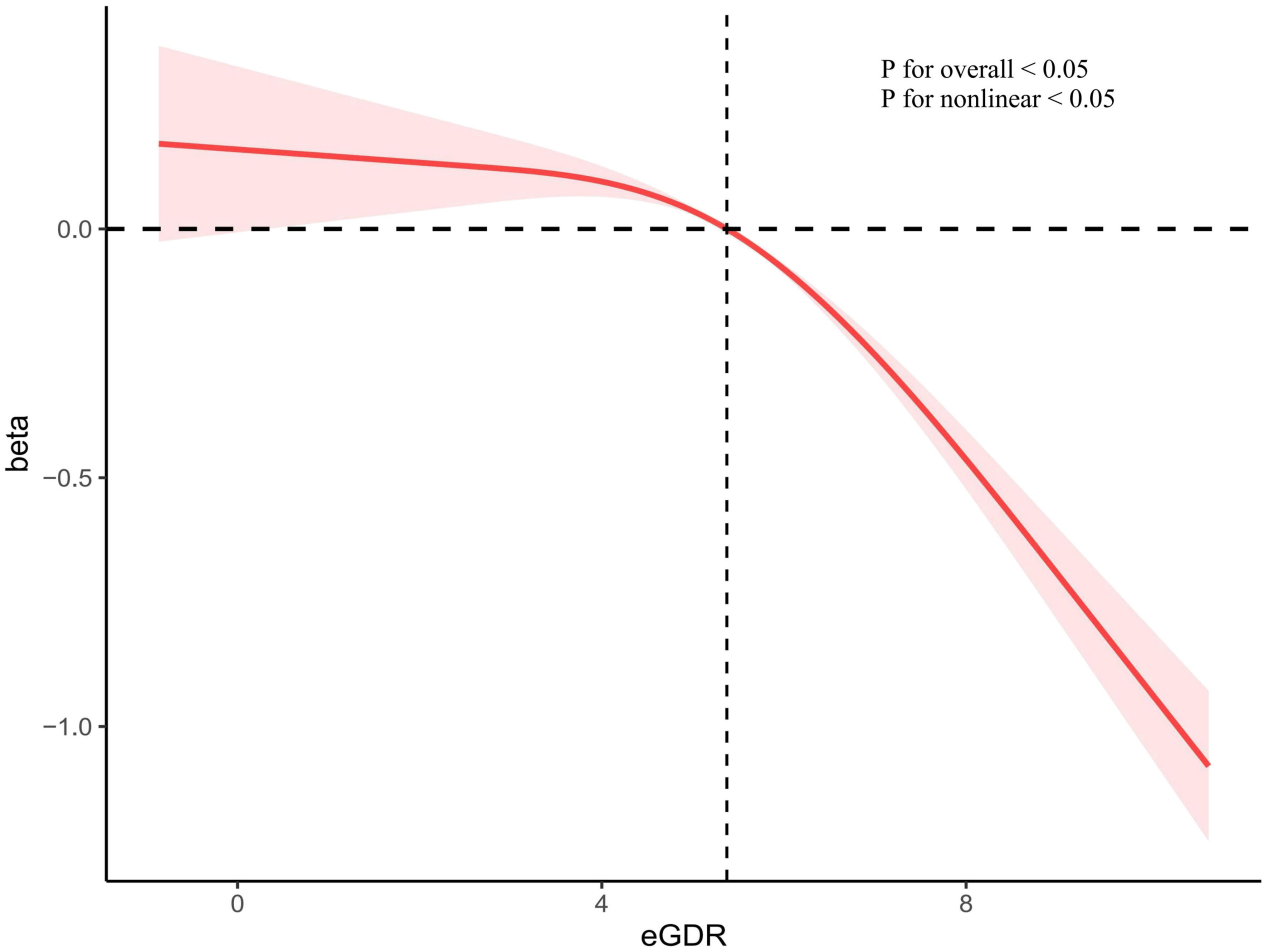
The correlation of eGDR with ePWV in a restricted cubic spline model. Adjusted for age, sex, race/ethnicity, education levels, smoking, BMI, TC, TG, eGFR, DM, CHD, CHF, Hyperlipidemia, anti-diabetic drugs and anti-hyperlipidemic drugs. eGDR, estimated glucose disposal rate; BMI, body mass index; TC, total cholesterol; TG, triglycerides; eGFR, estimated glomerular filtration rate; DM, diabetes mellitus; CHD, coronary heart disease; CHF, congestive heart failure.

In the unadjusted linear regression analysis, we observed a negative correlation between eGDR and ePWV (β=-0.24(-0.26–0.21, P< 0.001). In Model II, eGDR was significantly negatively correlated with ePWV (β=-0.13(-0.14–0.11, P< 0.001). However, TyG index and HOMA-IR showed no significant correlation to arterial stiffness (**Table 2**). Restricted cubic spline indicated a non-linear inverse relationship between eGDR and ePWV (P for nonlinear < 0.05). As eGDR increases, ePWV decreases more significantly (**Figure 2**).

**Table 2:** Beta between ePWV by eGDR in the NHANES 1999-2018.

	Non-adjusted model		Model I		Model II	
	Beta [95% CI]	*P* value	Beta [95% CI]	*P* value	Beta [95% CI]	*P* value
eGDR	-0.24 (-0.26, -0.21)	<0.001**	-0.08 (-0.10, -0.07)	<0.001**	-0.13 (-0.14, -0.11)	<0.001***
TyG	0.19 (0.10,0.28)	<0.001**	0.08 (0.02, 0.14)	0.01*	0.11 (-0.03, 0.25)	0.14
HOMA-IR	0.01 (0.00,0.01)	0.16	0 (0.00, 0.00)	0.82	0 (-0.01, 0.00)	0.30

Data are presented as Beta (95% CI). Model I adjusted for age, sex, and race/ethnicity. Model II adjusted for age, sex, race/ethnicity, education levels, smoking, BMI, TC, TG, eGFR, DM, CHD, CHF, Hyperlipidemia, anti-diabetic drugs and anti-hyperlipidemic drugs. eGDR, estimated glucose disposal rate; BMI, body mass index; TC, total cholesterol; TG, triglycerides; HOMA-IR, Homeostatic Model Assessment for Insulin Resistance; TyG, triglyceride and glucose index; ePWV, estimated pulse wave velocity; eGFR, estimated glomerular filtration rate; DM, diabetes mellitus; CHD, coronary heart disease; CHF, congestive heart failure; ***P value<0.001, **P value<0.01, *P value<0.05.

### Kaplan–Meier survival analysis curves for mortality based on eGDR

**Figure 3:**
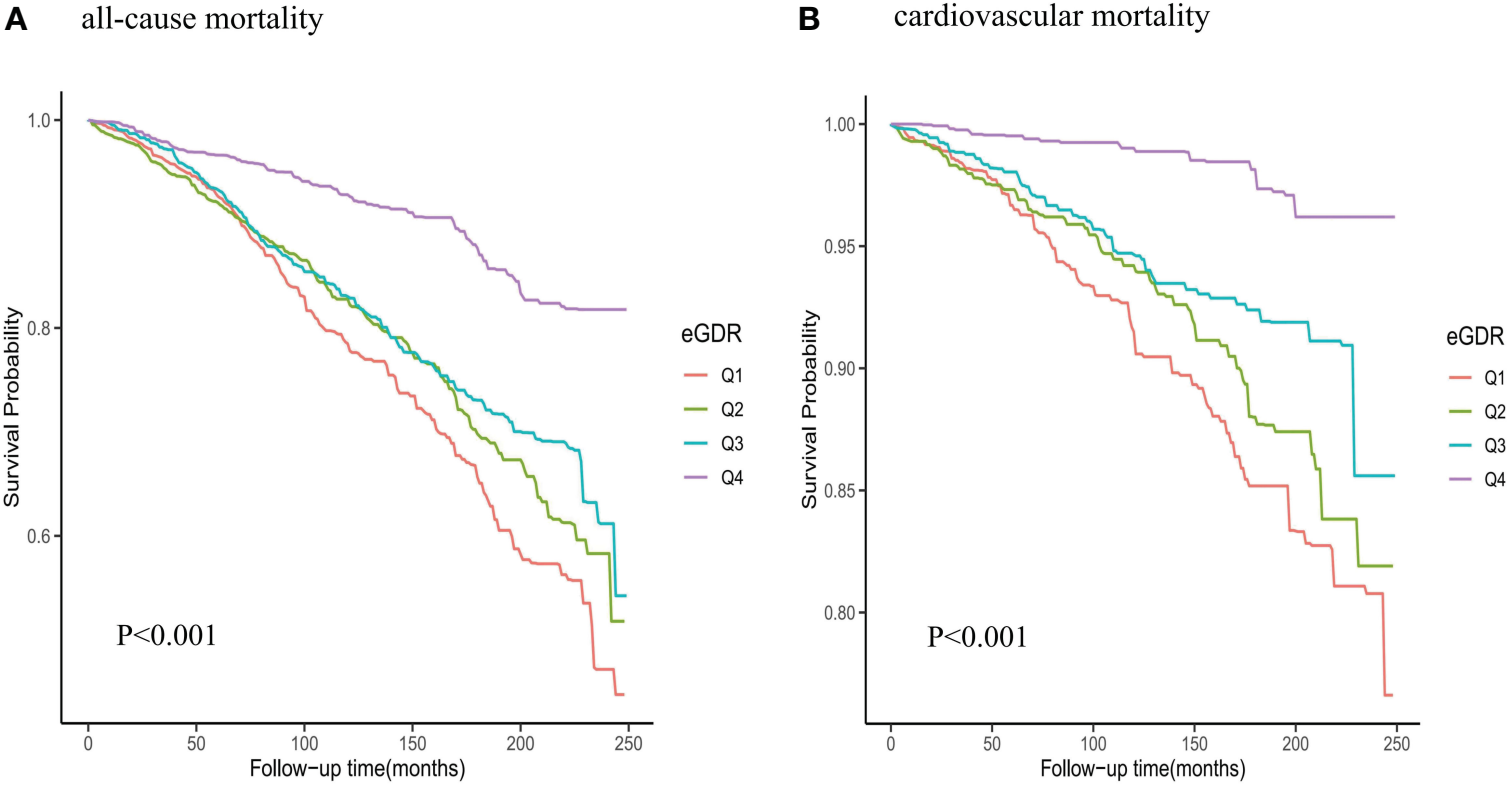
Kaplan-Meier survival analysis curves for all-cause and cardiovascular mortality among eGDR groups. **(A)** all-cause mortality; **(B)** cardiovascular mortality.

1051 all-cause deaths and 283 CVD deaths were showed during the follow-up period. The mortality rate of eGDR group was shown in the **Figure 3**. We observed significant differences in mortality between different eGDR groups (all-cause mortality: P <0.001; cardiovascular mortality: P <0.001). The all-cause mortality and cardiovascular mortality rates were significantly decreased in the higher eGDR group.

### Relationship between eGDR and mortality

We also used cox regression model to evaluate the association between eGDR and mortality. Represented as a continuous variable, we observed a negative correlation between eGDR and all-cause mortality with a hazard ratio (HR) of 0.93 (95%CI: 0.89-0.98) and cardiovascular mortality with a hazard ratio (HR) of 0.84 (95%CI: 0.77-0.92). Compared with participants having lowest eGDR, those having highest eGDR had a reduction of 35% (adjusted HR, 0.65; 95% CI, 0.48-0.89) in the risk for all-cause mortality and 65% (adjusted HR, 0.35; 95% CI, 0.19-0.65) in the risk for cardiovascular mortality in Model II (**Table 3**).

**Table 3:** Weighted cox regression analysis on the association between eGDR and mortality.

	Non-adjusted model		Model I		Model II	
	HR [95% CI]	*P* value	HR [95% CI]	*P* value	HR [95% CI]	*P* value
All-cause mortality						
Continuous eGDR	0.86 (0.83,0.89)	<0.001***	0.91 (0.88, 0.95)	<0.001***	0.93 (0.89,0.98)	0.01*
eGDR-Q1	Reference	–	Reference	–	Reference	–
eGDR-Q2	0.84 (0.68,1.04)	0.11	0.77 (0.62, 0.96)	0.02*	0.82 (0.64,1.06)	0.13
eGDR-Q3	0.76 (0.60,0.95)	0.02*	0.89 (0.71, 1.12)	0.33	0.89 (0.63,0.98)	0.36
eGDR-Q4	0.33 (0.25,0.42)	<0.001***	0.55 (0.42, 0.72)	<0.001***	0.65 (0.48,0.89)	0.01*
Cardiovascular mortality						
Continuous eGDR	0.78 (0.73,0.83)	<0.001***	0.81 (0.75, 0.88)	<0.001***	0.84 (0.77, 0.92)	<0.001**
eGDR-Q1	Reference	–	Reference	–	Reference	–
eGDR-Q2	0.79 (0.54,1.16)	0.23	0.74 (0.50, 1.10)	0.14	0.82 (0.53, 1.28)	0.39
eGDR-Q3	0.58 (0.37,0.92)	0.02*	0.72 (0.46, 1.11)	0.14	0.94 (0.55, 1.59)	0.81
eGDR-Q4	0.15 (0.09,0.26)	<0.001***	0.26 (0.16, 0.45)	<0.001***	0.35 (0.19, 0.65)	<0.001***

Data are presented as HR (95% CI). Model I adjusted for age, sex, and race/ethnicity. Model II adjusted for age, sex, race/ethnicity, education levels, smoking, BMI, TC, TG, eGFR, DM, CHD, CHF, Hyperlipidemia, anti-diabetic drugs and anti-hyperlipidemic drugs. eGDR, estimated glucose disposal rate; BMI, body mass index; TC, total cholesterol; TG, triglycerides; eGFR, estimated glomerular filtration rate; DM, diabetes mellitus; CHD, coronary heart disease; CHF, congestive heart failure; ***P value<0.001, **P value<0.01, *P value<0.05.

**Figure 4:**
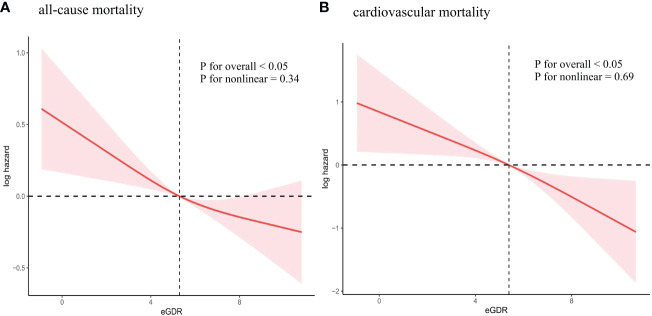
Association between eGDR and all-cause and cardiovascular mortality. **(A)** all-cause mortality; **(B)** cardiovascular mortality. Adjusted for age, sex, race/ethnicity, education levels, smoking, BMI, TC, TG, eGFR, DM, CHD, CHF, Hyperlipidemia, anti-diabetic drugs and anti-hyperlipidemic drugs. eGDR, estimated glucose disposal rate; BMI, body mass index; TC, total cholesterol; TG, triglycerides; eGFR, estimated glomerular filtration rate; DM, diabetes mellitus; CHD, coronary heart disease; CHF, congestive heart failure.

A restricted cubic spline was used to examine the association between eGDR and mortality. The findings indicated a linear inverse relationship between eGDR and mortality (all-cause mortality: P for non-linear=0.34; cardiovascular mortality: P for non-linear=0.69) (**Figure 4**). As eGDR rose, there was a substantial decrease in the risk of mortality (**Figure 4**).

### Sensitivity analysis

Similarly, sensitivity analysis adopting unweighted logistic analysis reveals that the lower risk of mortality was showed in the highest eGDR (all-cause mortality: HR = 0.61, 95%CI: 0.48-0.77; cardiovascular mortality: HR = 0.34, 95%CI: 0.20-0.57) in Model II (**Table 4**). These results suggest a consistent inverse relationship between eGDR and mortality.

**Table 4:** Unweighted cox regression analysis on the association between eGDR and mortality in sensitive analysis.

	Non-adjusted model		Model I		Model II	
	HR [95% CI]	*P* value	HR [95% CI]	*P* value	HR [95% CI]	*P* value
All-cause mortality						
Continuous eGDR	0.86(0.84,0.88)	<0.001***	0.92(0.90, 0.95)	<0.001***	0.92(0.89,0.96)	<0.001***
eGDR-Q1	Reference	–	Reference	–	Reference	–
eGDR-Q2	0.83(0.71,0.97)	0.02*	0.75(0.64, 0.88)	0.003**	0.74(0.63,0.88)	<0.001***
eGDR-Q3	0.75(0.63,0.87)	<0.001***	0.87(0.74, 1.03)	0.10	0.87(0.72,1.05)	0.14
eGDR-Q4	0.34(0.28,0.41)	<0.001***	0.58(0.47, 0.71)	<0.001***	0.61(0.48,0.77)	<0.001***
Cardiovascular mortality						
Continuous eGDR	0.79(0.75,0.83)	<0.001***	0.84(0.79, 0.89)	<0.001***	0.85(0.79, 0.91)	<0.001**
eGDR-Q1	Reference	–	Reference	–	Reference	–
eGDR-Q2	0.79(0.59,1.05)	0.11	0.73(0.55, 0.97)	0.03*	0.74(0.55, 1.01)	0.06
eGDR-Q3	0.60(0.44,0.81)	<0.001***	0.74(0.54, 1.01)	0.06	0.79(0.55, 1.14)	0.21
eGDR-Q4	0.16(0.10,0.25)	<0.001***	0.30(0.19, 0.48)	<0.001***	0.34(0.20, 0.57)	<0.001***

Data are presented as HR (95% CI). Model I adjusted for age, sex, and race/ethnicity. Model II adjusted for age, sex, race/ethnicity, education levels, smoking, BMI, TC, TG, eGFR, DM, CHD, CHF, Hyperlipidemia, anti-diabetic drugs and anti-hyperlipidemic drugs. eGDR, estimated glucose disposal rate; BMI, body mass index; TC, total cholesterol; TG, triglycerides; eGFR, estimated glomerular filtration rate; DM, diabetes mellitus; CHD, coronary heart disease; CHF, congestive heart failure; ***P value<0.001, **P value<0.01, *P value<0.05.

### ROC curve analysis of eGDR, TyG index and HOMA−IR

**Figure 5:**
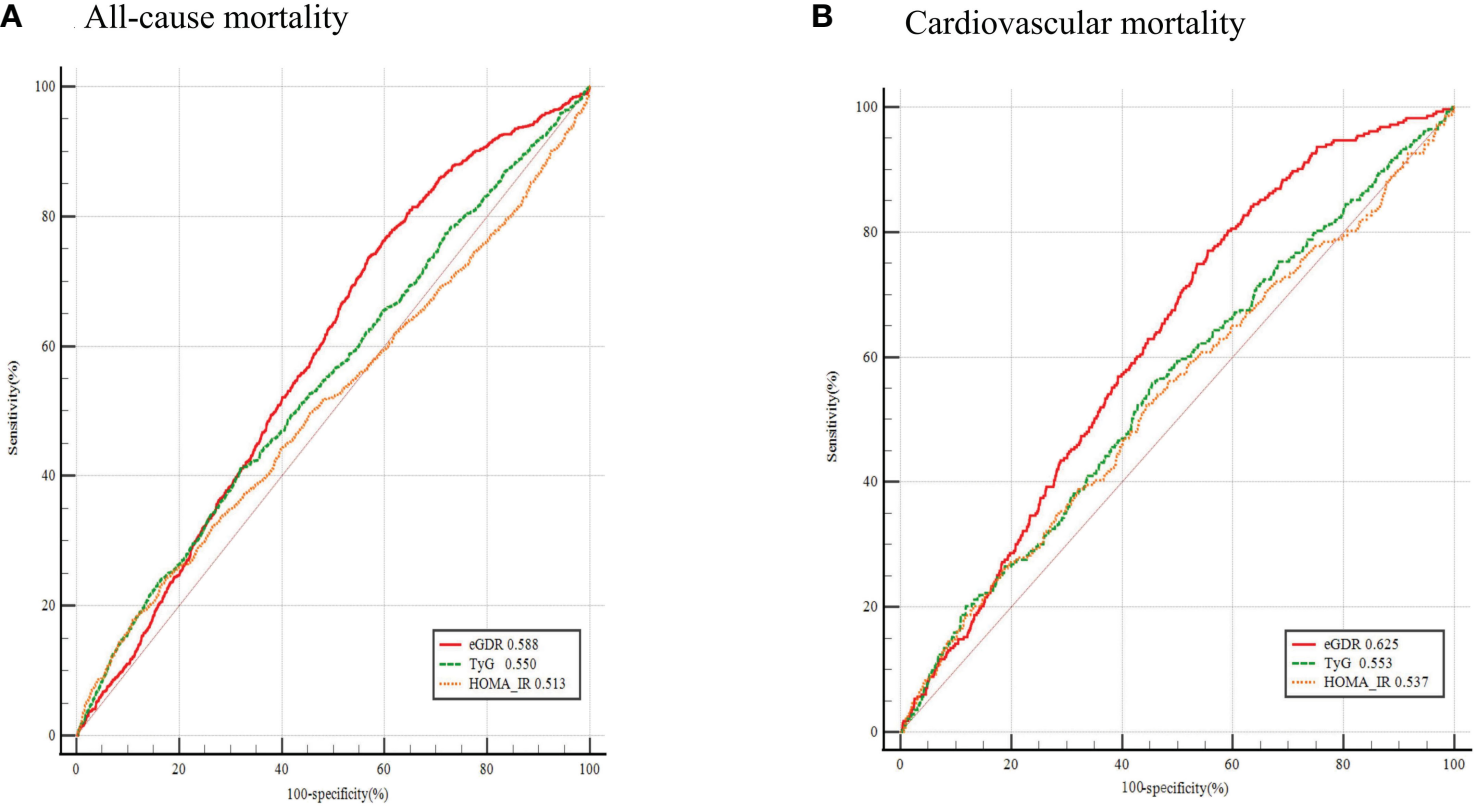
ROC Curve analysis for eGDR, TyG index and HOMA-IR Predicted all-cause and cardiovascular mortality. **(A)** All-cause mortality; **(B)** Cardiovascular mortality. ROC receiver operating characteristic; eGDR, estimated glucose disposal rate; TyG index triglyceride glucose index; HOMA-IR homeostasis model assessment of insulin resistance.

The ROC curves of eGDR, TyG index, and HOMA-IR predicting mortality in NAFLD patients are shown in **Table 5** and **Figure 5**. Compared with TyG index and HOMA-IR, the eGDR shows excellent predictive value in all-cause mortality (0.588 vs. 0.550 vs. 0.513, P < 0.001) and cardiovascular mortality (0.625 vs. 0.553 vs. 0.537, P < 0.001). In predicting all-cause mortality, its AUC was 0.588 (0.574,0.602) and the optimal cut-off value was 5.95. The sensitivity was 73.64 and the specificity was 53.15. In predicting cardiovascular mortality, its AUC was 0.625 (0.611,0.639) and the optimal cut-off value was 5.70. The sensitivity was 77.00 and the specificity was 54.50.

**Table 5:** ROC curves analysis on the association between IR indicators and mortality.

IR indicators	Best thresholds	Sensitivity	Specificity	AUC (95% CI)	*P* for difference
All-cause mortality					
eGDR	5.95	73.64	53.15	0.588(0.574,0.602)	Reference
TyG	7.70	41.3	67.9	0.550(0.536,0.564)	0.002**
HOMA-IR	11.33	17.8	89.1	0.513(0.498,0.527)	<0.001***
Cardiovascular mortality					
eGDR	5.70	77.00	54.50	0.625(0.611,0.639)	Reference
TyG	7.51	55.80	54.60	0.553(0.538,0.567)	<0.001***
HOMA-IR	5.64	52.30	55.60	0.537(0.523,0.551)	<0.001***

ROC, receiver operating characteristic; IR, insulin resistance; eGDR, estimated glucose disposal rate; TyG, triglyceride-glucose index; HOMA-IR, homeostasis model assessment of insulin resistance; ***P value<0.001, **P value<0.01, *P value<0.05.

### Subgroups analysis

**Figure 6:**
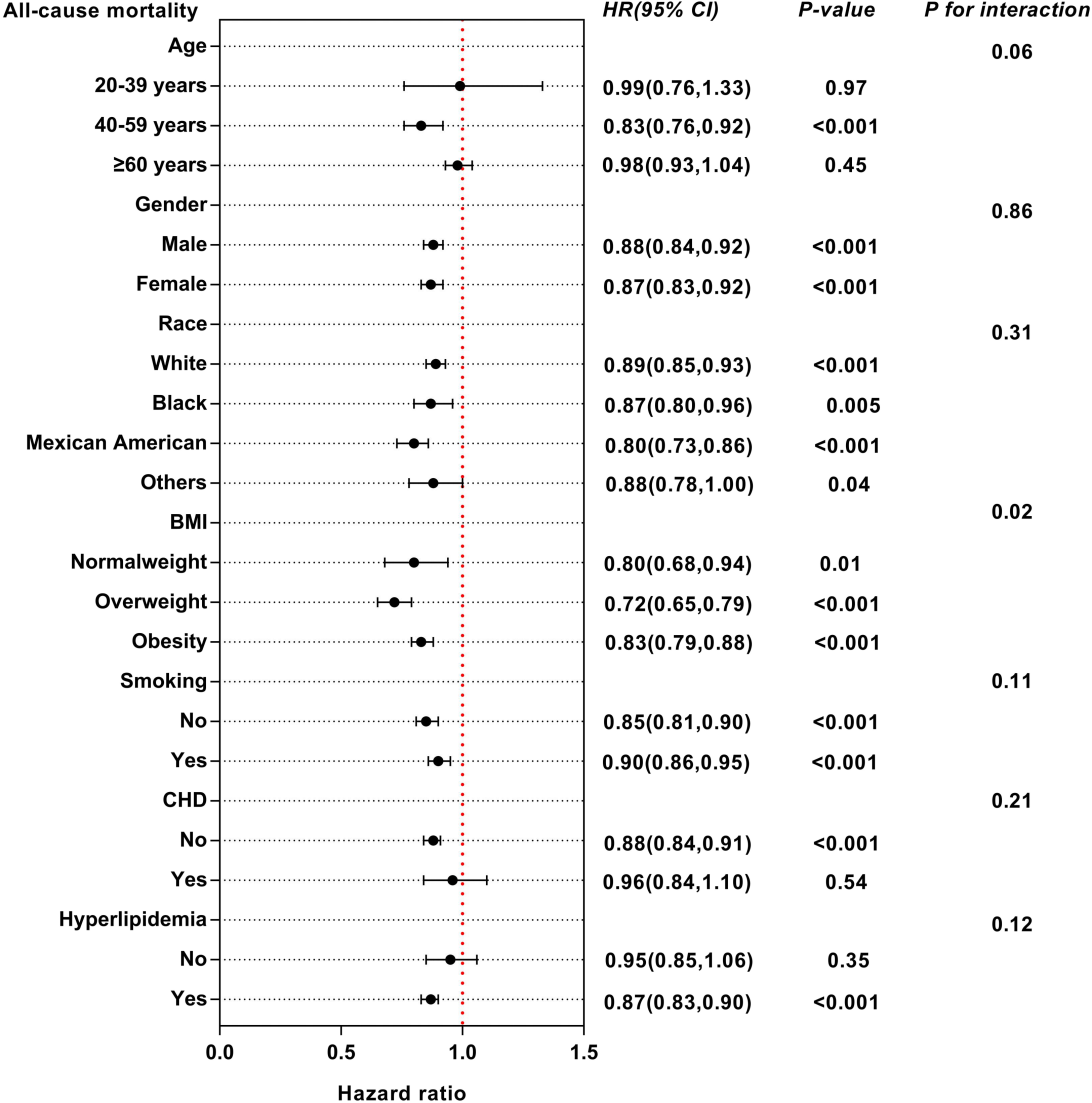
Subgroup analysis of multi-variable adjusted association of eGDR with all-cause mortality.

**Figure 7:**
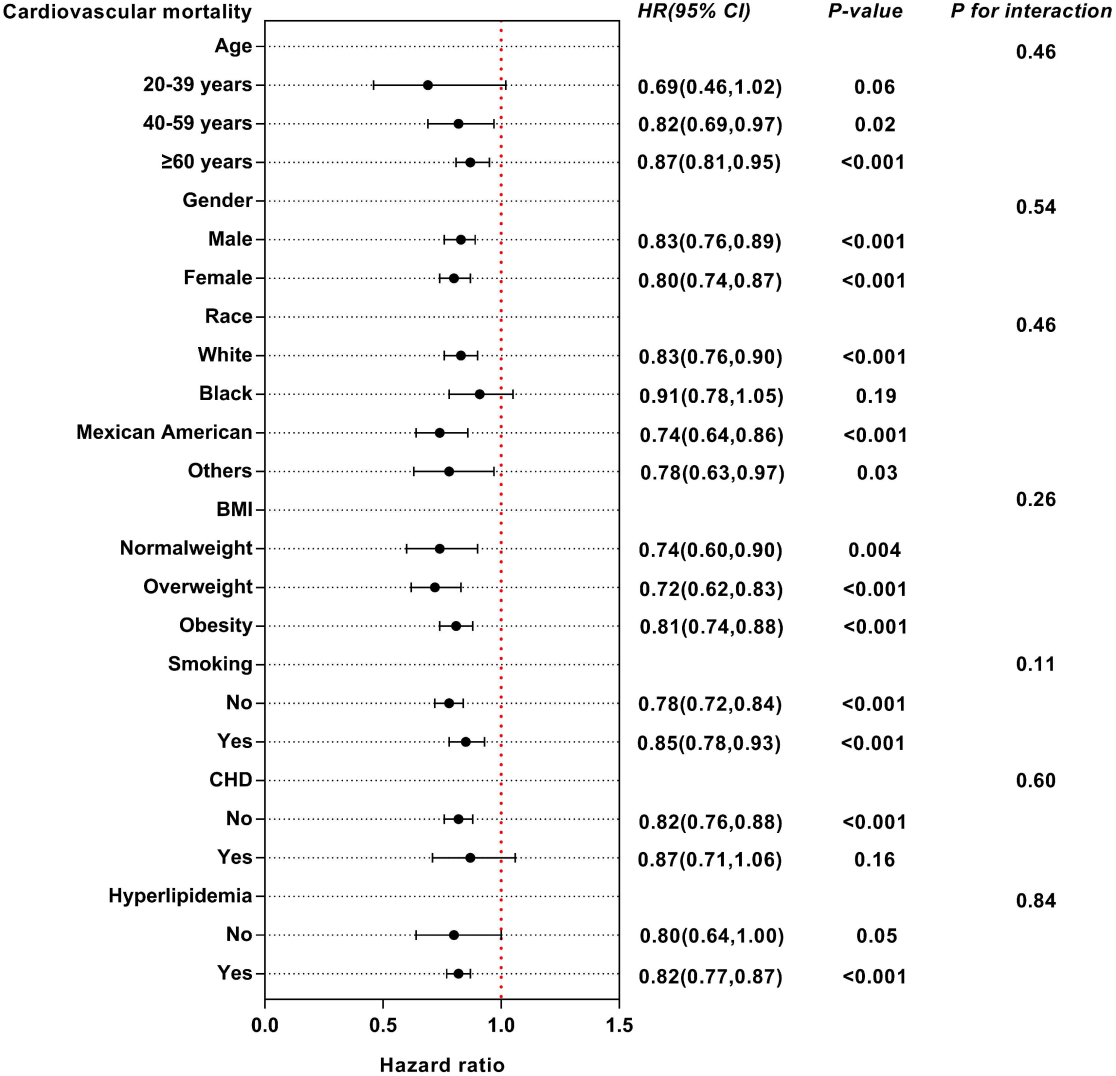
Subgroup analysis of multi-variable adjusted association of eGDR with cardiovascular mortality.

We conducted subgroup analysis to examine the possible link between eGDR and mortality among diverse subgroups categorized by age, sex, race, BMI, smoking, CHD and hyperlipidemia (**Supplementary Tables S1**, **S2**). For all-cause mortality, eGDR may have interactive effects in different BMI populations (**Figure 6**). The influence of eGDR on cardiovascular mortality did not vary among the subgroups (**Figure 7**).

## Discussion

Previous studies have found that low eGDR is associated with the increased risk of prevalence and poor prognosis in various diseases. Specifically, our findings demonstrate that the eGDR was inversely associated with the all-cause mortality and cardiovascular mortality after accounting for confounding factors in the adult population of the United States. It performs better than the TyG index and HOMA-IR in predicting these outcomes. The relationship between eGDR and all-cause as well as cardiovascular mortality follows a linear pattern, as depicted by the fitted smoothing curves. Interestingly, the effect of eGDR on cardiovascular mortality does not differ significantly among different subgroups. For all-cause mortality, eGDR may have interactive effects in different BMI populations. In addition, we found a significant negative correlation between eGDR and arterial stiffness. However, TyG index and HOMA-IR showed no significant correlation to arterial stiffness.

Previous studies have shown that IR is common in diabetes patients, and severe IR is positively related to poor prognosis (31). Many studies have shown that IR also plays an important role in other diseases, including hypertension, NAFLD, CHF, etc (32–34). We also found that IR plays an important role in NAFLD. Additionally, research suggests that an intricate interplay between metabolic elements, adipose tissue breakdown and IR leads to a harmful progression that could connect fatty liver disease with severe cardiovascular disease. The difference from previous studies is that the severity of IR can also predict poor prognosis in NAFLD. Furthermore, individuals diagnosed with fatty liver disease face an increased likelihood of atherosclerosis, adverse cardiovascular events and higher mortality (35). Previous studies have shown that NAFLD is linked to increased levels of IR, a significant pathophysiological factor that plays a role in the onset and advancement of the disease (36). In addition, the elastic fibers in the inner layer of the artery undergo degeneration and the intima becomes hard, which can lead to an increase in arterial hardness (37). Arterial stiffness is considered an independent risk predictor of cardiovascular events (38). IR can damage the endothelium of blood vessels and lead to inflammatory reactions, which may easily lead to arterial stiffness and arteriosclerosis (39). A Meta-analysis of 37,780 Individuals showed that IR is closely related to arterial stiffness (40). Another study suggests that the TyG index is closely related to arterial stiffness in uncontrolled hypertensive patients in American adults (41). In addition, we found that low eGDR levels are related to an increased risk of arterial stiffness in NAFLD patients in our study. This indicates that IR also plays an important role in arteriosclerosis in NAFLD.

IR is related to various factors such as inflammation, oxidative stress, microRNA expression, abnormal insulin metabolism signaling pathways and mitochondrial dysfunction in the body (42). IR is also an important characteristic of NAFLD. Therefore, the evaluation of IR indicators is closely associated with the prognosis of NAFLD patients. Inflammation and oxidative stress are closely related to IR. Recently, many studies found that high levels of inflammation and oxidative stress both lead to high mortality rates in patients with fatty liver disease (43, 44). Furthermore, the eGDR and TyG index have been proven to be a simple indicator for evaluating IR. Based on these findings, our results indicate that low eGDR levels are related to an increased risk of all-cause mortality and cardiovascular mortality in NAFLD patients in the United States. And the predictive ability of eGDR on outcomes is superior to TyG index and HOMA-IR.

In addition, the NHANES data is designed through complex, multi-stage probability sampling to ensure the robustness of the results. In subgroup analysis, eGDR has a certain predictive effect on all-cause mortality in different BMI populations especially in overweight populations. It may be due to the increased risk of IR in overweight or obese populations. The effect of eGDR on cardiovascular mortality does not differ significantly among different subgroups. These are points worth paying attention to in our study.

This research demonstrates, for the first time, the relationship between eGDR and arterial stiffness and mortality. These findings can provide important reference value for the prognosis of patients with NAFLD in the adult population of the United States. This study utilized a large sample of national databases and had a long follow-up time, which enhances the credibility of the research findings. However, there are some limitations in our study. Firstly, this study did not monitor the dynamic changes of eGDR, which may provide greater reference value. Second, the findings of the NHANES study are primarily applicable to the American population because of the variations in disease characteristics across different racial groups. Finally, the diagnosis of NAFLD mainly relies on us-FLI, which may lead to selection bias. Therefore, future research needs to consider these limitations.

## Conclusion

Low eGDR (an indicator of insulin resistance) levels are related to an increased risk of arterial stiffness and mortality in NAFLD patients in the United States. However, further prospective studies are still needed to reveal their relationship.

## Data availability statement

The original contributions presented in the study are included in the article/supplementary material. Further inquiries can be directed to the corresponding authors.

## Author contributions

JS: Writing – original draft. RM: Writing – review & editing. LY: Writing – review & editing.
