# Supplementary material for: Associations between estimated glucose disposal rate and arterial stiffness and mortality among US adults with non-alcoholic fatty liver disease
Source: Front Endocrinol (Lausanne). 2024 May 8;15:1398265. doi: 10.3389/fendo.2024.1398265 (PMC11109450; doi:10.3389/fendo.2024.1398265)
Supplement: Supplementary file 1 [file Table_1.docx]

**Table S1: Subgroup analysis of multi-variable adjusted association of eGDR with the risk of all-cause mortality.**

| Variable name | Without all-cause mortality | All-cause mortality | p value | p for interaction |
| --- | --- | --- | --- | --- |
|  |  |  |  | 0.06 |
| Age 20-39 years | ref | 0.99(0.76,1.33) | 0.97 |  |
| Age 40-59 years | ref | 0.83(0.76,0.92) | <0.001 |  |
| Age≥60 years | ref | 0.98(0.93,1.04) | 0.45 |  |
|  |  |  |  | 0.86 |
| Gender-Male | ref | 0.88(0.84,0.92) | <0.001 |  |
| Gender-Female | ref | 0.87(0.83,0.92) | <0.001 |  |
|  |  |  |  | 0.31 |
| Race-White | ref | 0.89(0.85,0.93) | <0.001 |  |
| Race-Black | ref | 0.87(0.80,0.96) | 0.005 |  |
| Race-Mexican American | ref | 0.80(0.73,0.86) | <0.001 |  |
| Race-Others | ref | 0.88(0.78,1.00) | 0.04 |  |
|  |  |  |  | 0.02 |
| BMI-Normalweight | ref | 0.80(0.68,0.94) | 0.01 |  |
| BMI-Overweight | ref | 0.72(0.65,0.79) | <0.001 |  |
| BMI-Obesity | ref | 0.83(0.79,0.88) | <0.001 |  |
|  |  |  |  | 0.11 |
| No-Smoking | ref | 0.85(0.81,0.90) | <0.001 |  |
| Smoking | ref | 0.90(0.86,0.95) | <0.001 |  |
|  |  |  |  | 0.21 |
| Non-CHD | ref | 0.88(0.84,0.91) | <0.001 |  |
| CHD | ref | 0.96(0.84,1.10) | 0.54 |  |
|  |  |  |  | 0.12 |
| Non-Hyperlipidemia | ref | 0.95(0.85,1.06) | 0.35 |  |
| Hyperlipidemia | ref | 0.87(0.83,0.90) | <0.001 |  |

Continuous data were presented as the mean and 95% confidence interval, category data were presented as the proportion and 95% confidence interval. BMI, body mass index; CHD, coronary heart disease.
