# Supplementary material for: Associations between estimated glucose disposal rate and arterial stiffness and mortality among US adults with non-alcoholic fatty liver disease
Source: Front Endocrinol (Lausanne). 2024 May 8;15:1398265. doi: 10.3389/fendo.2024.1398265 (PMC11109450; doi:10.3389/fendo.2024.1398265)
Supplement: Supplementary file 2 [file Table_2.docx]

**Table S2: Subgroup analysis of multi-variable adjusted association of eGDR with the risk of cardiovascular mortality.**

| Variable name | Non-cardiovascular mortality | Cardiovascular mortality | p value | p for interaction |
| --- | --- | --- | --- | --- |
|  |  |  |  | 0.46 |
| Age 20-39 years | ref | 0.69(0.46,1.02) | 0.06 |  |
| Age 40-59 years | ref | 0.82(0.69,0.97) | 0.02 |  |
| Age≥60 years | ref | 0.87(0.81,0.95) | <0.001 |  |
|  |  |  |  | 0.54 |
| Gender-Male | ref | 0.83(0.76,0.89) | <0.001 |  |
| Gender-Female | ref | 0.80(0.74,0.87) | <0.001 |  |
|  |  |  |  | 0.46 |
| Race-White | ref | 0.83(0.76,0.90) | <0.001 |  |
| Race-Black | ref | 0.91(0.78,1.05) | 0.19 |  |
| Race-Mexican American | ref | 0.74(0.64,0.86) | <0.001 |  |
| Race-Others | ref | 0.78(0.63,0.97) | 0.03 |  |
|  |  |  |  | 0.26 |
| BMI-Normalweight | ref | 0.74(0.60,0.90) | 0.004 |  |
| BMI-Overweight | ref | 0.72(0.62,0.83) | <0.001 |  |
| BMI-Obesity | ref | 0.81(0.74,0.88) | <0.001 |  |
|  |  |  |  | 0.11 |
| No-Smoking | ref | 0.78(0.72,0.84) | <0.001 |  |
| Smoking | ref | 0.85(0.78,0.93) | <0.001 |  |
|  |  |  |  | 0.60 |
| Non-CHD | ref | 0.82(0.76,0.88) | <0.001 |  |
| CHD | ref | 0.87(0.71,1.06) | 0.16 |  |
|  |  |  |  | 0.84 |
| Non-Hyperlipidemia | ref | 0.80(0.64,1.00) | 0.05 |  |
| Hyperlipidemia | ref | 0.82(0.77,0.87) | <0.001 |  |

Continuous data were presented as the mean and 95% confidence interval, category data were presented as the proportion and 95% confidence interval. BMI, body mass index; CHD, coronary heart disease.
